# Telework at times of a pandemic: The role of voluntariness in the perception of disadvantages of telework

**DOI:** 10.1007/s12144-022-03047-5

**Published:** 2022-04-01

**Authors:** Antonia J. Kaluza, Rolf van Dick

**Affiliations:** Department of Psychology, Goethe University Frankfurt, Theodor-W.-Adorno-Platz 6, 60323 Frankfurt, Germany

**Keywords:** Telework, Telecommuting, Voluntariness, COVID-19, Disadvantages

## Abstract

The implications of telework are discussed controversially and research on its positive and negative effects has produced contradictory results. We explore voluntariness of employee telework as a boundary condition which may underpin these contradictory findings. Under normal circumstances, individuals who do more telework should perceive fewer disadvantages. However, during the COVID-19 pandemic, employees could no longer voluntarily choose to telecommute, as many organizations were forced to introduce telework by governmental regulations. In two studies, we examine whether the voluntary nature of telework moderates the association between the amount of telework and perceptions of disadvantage. In Study 1, we collected data before and during the COVID-19 pandemic (*N* = 327). Results show that pre-pandemic participants (who were more likely to voluntarily choose this form of work) reported fewer disadvantages the more telework they did, but this was not the case for employees during the COVID-19 pandemic. To validate these findings, we measured employees’ voluntariness of telework in Study 2 (*N* = 220). Results support the importance of voluntariness: Individuals who experience a high degree of voluntariness in choosing telework perceive fewer disadvantages the more they telework. However, the amount of telework was not related to reduced perceptions of disadvantages for those who experienced low voluntariness regarding the telecommuting arrangement. Our findings help to understand when telework is related to the perception of disadvantages and they can provide organizations with starting points for practical interventions to reduce the negative effects of telework.

## Introduction

The COVID-19 pandemic has brought about many changes in almost all areas, including in the way we work. Since March 2020, the majority of organizations have switched to telework, either in full or for several days a week to reduce the physical presence of employees at the workplace and thus reduce the possibility of infections. These changes have been accompanied by reduced face-to-face interaction and, as a possible result, increased feelings of isolation.

Teleworking, i.e. work activities performed outside a company-based workplace (mainly from home), where technologies are used to interact with colleagues or customers and to complete work tasks (e.g., Allen et al., 2015), has advantages and disadvantages (see e.g., Gajendran & Harrison, 2007). Previous studies have produced contradictory results revealing both positive and negative consequences of telecommuting (e.g., Golden & Eddleston, 2020; Vega et al., 2015) such as increased job satisfaction, improved work-life balance, higher productivity, or reduced costs for organizations in some studies whereas other studies found lower work satisfaction, increased work-family conflicts or difficulties in developing shared knowledge in teams (see Boell et al., 2016 for an overview).

Under normal circumstances, it can be assumed that those employees are more likely to telecommute who prefer this type of work because they appreciate its advantages (e.g., higher autonomy, more flexibility) and perceive less disadvantages (e.g., blurred boundaries between work and non-work, social isolation). However, if an employer prescribes telework—as during the COVID-19 pandemic all over the world across many occupations both in the public and private sector—employees are forced to perform telework, regardless of their own preferences. This raises the question whether the involuntary nature of telework affects employees’ perception of negative consequences of telework, such as feelings of isolation or work-life conflicts. Drawing on theory and recent empirical findings, we propose that those individuals who voluntarily choose this form of work will perceive fewer disadvantages of telework the more they make use of this alternative work arrangement but that this will not be the case for those individuals who did not initiate telework by themselves (as was the case during the COVID-19 pandemic in many organizations).

In two studies, we examine voluntariness of telework as a boundary condition and we predict that it moderates the relation between the amount of telework and the perception of disadvantages. By doing so, we seek to make two contributions to the extant telework literature. First, previous studies on teleworking have mainly looked at the positive and negative sides of teleworking and have produced contradictory results, documenting a lack of research on potential boundary conditions, especially the voluntariness of telework. Even though the important role of voluntariness has been discussed theoretically (e.g., Allen et al., 2013), there is little empirical research examining the voluntary nature of this working form (for an exception, see e.g., Lapierre et al., 2016). Rather than revisiting the question of whether telework is positive or negative, we thus follow Boell et al.’s (2016) call and turn to the question of when and under what conditions telework—or more specifically the amount of telework—is associated with the perception of more disadvantages. Second, we combine 1) a field study that utilizes the relatively sudden and widespread introduction of telework in Germany due to the COVID-19 pandemic with 2) a survey study asking more specifically about the voluntariness of telework. In our first study, we divided participants into two groups: The first group was surveyed before the introduction of COVID-related restrictions which made them more likely to choose telework voluntarily, while the second group was surveyed after many organizations in Germany encouraged and/or required their employees to work from home and thus, they are more likely to telework due to the circumstances and not their own preferences. By complementing this first field study with a second study in which we asked participants more directly about the voluntariness of telecommuting in their organization, we can thus draw more detailed conclusions and hence extend previous research.

### Theory and Hypotheses Development

Telecommuting, also often referred to as telework, remote work, or distributed work, has been defined by Gajendran and Harrison (2007), as “an alternative work arrangement in which employees perform tasks elsewhere that are normally done in a primary or central workplace, for at least some portion of their work schedule, using electronic media to interact with others inside and outside the organization” (p. 1525). Teleworking does not necessarily have to be full-time but can also be limited to certain time periods or parts of the work. In other words, the intensity and frequency of telework may vary (e.g., Gajendran et al., 2015). It is often discussed that it is not enough to simply distinguish between telecommuters and non-telecommuters, but that the amount of telework would be critical (Allen et al., 2015). Golden and Veiga (2005), for instance, found a curvilinear relation between the amount of telework and job satisfaction. In a sample of 321 employees, they found a linear increase of job satisfaction with more telework but only up to a point whereas at more extensive levels of telework job satisfaction reached a plateau. In a further study, Golden et al. (2008) identified feelings of social isolation increasing with the amount of telework and that this was related to lower job performance in a sample of 261 professional-level employees. So, someone who only works from home a few hours per week will encounter different experiences and consequences than someone who works remotely most of the week (Gajendran & Harrison, 2007). The question of whether teleworking is positive or negative is a matter of both diverging opinions and contradictory research findings (Allen et al., 2003; Boell et al., 2016). Previous studies have revealed both positive and negative consequences, which Gajendran and Harrison (2007) have called the “telecommuting paradox” (p. 1526; see also Boell et al., 2016). Social isolation is often seen as one of the main challenges of telecommuting (e.g., Allen et al., 2015), as employees working away from the organization’s premises have less personal face-to-face contact with their colleagues, which makes communication and exchange of ideas and support more difficult. If telecommuters spend less time with their colleagues, there is a risk that they will feel less integrated within their workgroup (Morganson et al., 2010), that they may develop poorer relationships with their colleagues and direct supervisors and become less involved in information exchange in organizations (e.g., Allen et al., 2015; Fonner & Roloff, 2010). In addition, telework may intensify work–family interference (e.g., Golden et al., 2006; Lapierre & Allen, 2006). As argued by Golden et al. (2006), telecommuters tend to be more present and available at home and may therefore be more involved in family issues and hence, interrupted in their work activities. At the same time, there is also a risk that telecommuters work overtime and have difficulties in disengaging from work (Eddleston & Mulki, 2017).

However, these negative effects have not been found in every study (e.g., Allen et al., 2015; Golden et al., 2006). It is thus likely that the perception of negative effects of telework will depend on other factors. We suggest that one such boundary condition and explanation for the inconsistent findings from prior research could be voluntariness or an employee’s control over the telecommuting arrangement (as argued by Allen et al., 2013). In case of voluntarily chosen telework, employees can decide for themselves to what extent they wish to make use of this alternative form of working, which can increase the perceived autonomy and should therefore be accompanied by a reduced perception of negative consequences, as proposed by Gajendran and Harrison (2007). This pattern is in line with the predictions of Festinger’s theory of cognitive dissonance (Festinger, 1957) which proposes that people adapt their cognitions to the circumstances in order to reduce dissonance, since dissonant states are perceived as unpleasant. For telework this would mean that people “rationalize away” the negative aspects that they experience when teleworking to avoid inconsistencies in their cognitive system that would otherwise occur when they simultaneously hold the cognitions of “I do a lot of telework” and “telework comes with many disadvantages.” However, the basic premise of the theory of cognitive dissonance is that people need to perceive having free choice of engaging in the behavior or not. If people are forced (by others or the circumstances), no dissonance occurs as external factors provide justification for the inconsistencies. Thus, when employees telework voluntarily, they are less likely to perceive disadvantages of such a working form and more likely to suppress negative aspects in order to avoid dissonance.

Likewise, the job demands control model predicts that employees cope better with potential negative aspects of job demands when they feel they have control over their work (Karasek, 1979, 2004). That is, control or decision latitude should mitigate the negative effects of work demands, since such a job offers learning and development potential for employees and is thus perceived as less stressful. Telework may increase feelings of loneliness (Golden et al., 2008), bears the risk of distractions in the home environment or the lack of adequate job-related resources (Greer & Payne, 2014), or might lead to exhaustion due to overwork or family-work conflict (see Boell et al., 2016). In line with the job demand control model, we believe that such negative effects are mainly experienced by those who feel forced to work from home (i.e. perceive low control), whereas those who can decide if and how much they work outside the work premises would feel psychological ownership and suffer less from potential downsides.

Finally, there is also initial empirical evidence for assuming a moderating role of voluntariness. Lapierre and colleagues (2016) conducted a longitudinal field study of sales people and found that those who had been forced to telework reported more strain-based work-to-family conflict. Similarly, employees who did not work remotely due to their own choice reported lower turnover intentions than those who did not telework due to organizational restrictions (Choi, 2018). Finally, Meyer et al. (2021) found in a three-wave study during the COVID-19 pandemic that women with children who worked from home while childcare was unavailable were exhausted particularly strongly but that autonomy mitigated this relation.

**Fig. 1:**
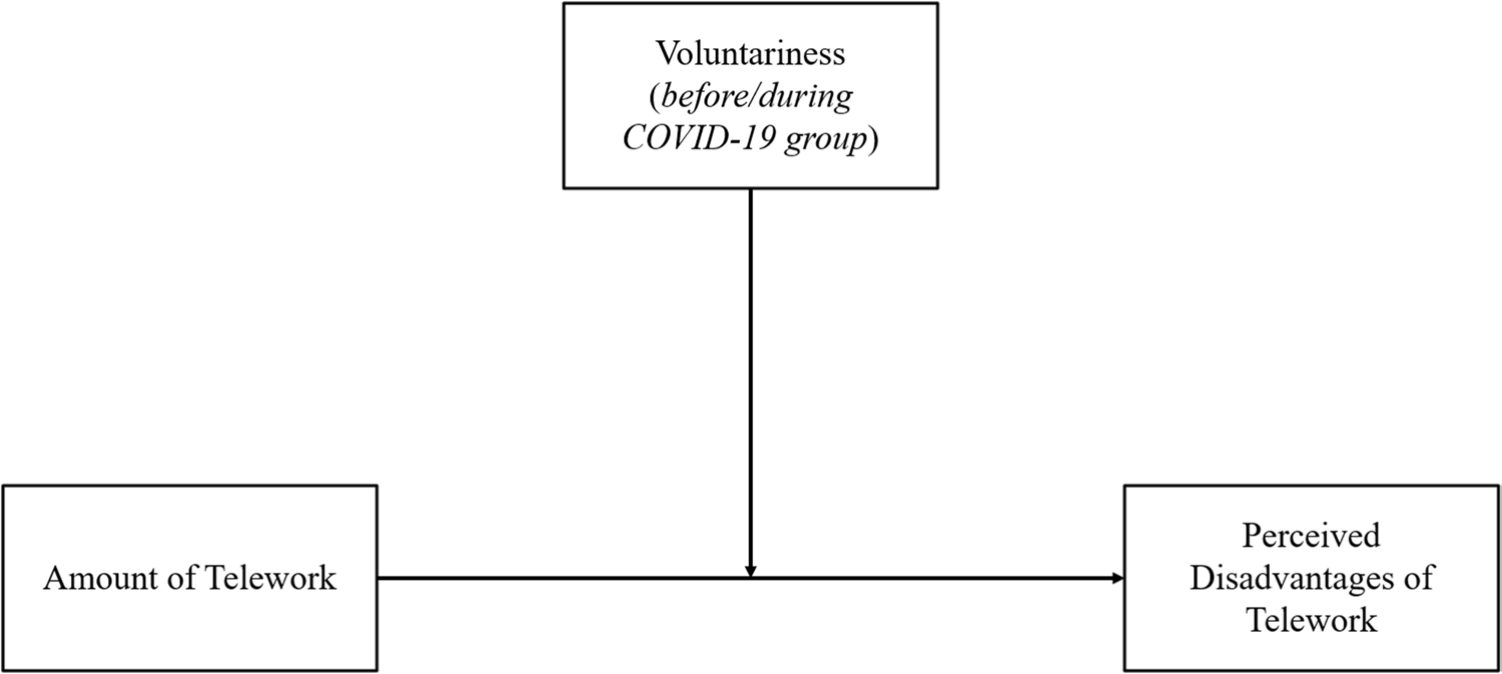
Theoretical Model

These findings support the notion that employees who can voluntarily choose to telework may perceive less disadvantages the more they telework compared to employees who cannot voluntarily choose whether to telework or not. Hence, we predict voluntariness of telework as a moderating factor (see Fig. 1):

#### Hypothesis

Voluntariness of telework serves as a boundary condition in that it moderates the association between the amount of telework and perceived disadvantages. More, specifically, the amount of telework will be associated with lower perceptions of disadvantages if the employee has chosen telework voluntarily but there will be no association between amount of telework and perceived disadvantages for employees who did not choose telework voluntarily.

## Overview of the Current Research

We conducted two studies to test our hypothesis. In the first study, we collected data before and during the COVID-19 pandemic, when many organizations required their employees to do telework, regardless of their preferences. We used March 19, 2020, as the cut-off date, as this was the week when far-reaching restrictions of social contacts were introduced in Germany. This included the “obligation for companies to organize working from home for every position where this is possible” (European Commission, 2020, p.11). We chose this date because we included new items in our questionnaire from this date onward, for example, to capture changes in telework. We assumed that people who filled out the survey after this date would – on average – work from home less voluntarily than those before the pandemic. Our cut-off date for separating the two groups is in line with other research on telework in Germany that has identified March/April 2020 as the time of a spark in telework (see Gollwitzer et al., 2021; Kluck et al., 2021; Meyer et al., 2021). To more directly test whether the voluntariness of telecommuting would explain differences in perceived disadvantages, we then conducted a second cross-sectional survey study in which we explicitly measured the voluntariness of telecommuting.

## Study 1

### Method

#### Participants and Procedure

We collected data from employees working in different organizations and branches in Germany. Participants were recruited online via social networks (e.g., Facebook, LinkedIn) and through the authors’ personal networks. Participation was voluntary, no compensation was paid for participation and participants were assured that their responses would be handled confidentially. A total of 331 people completed the questionnaire, four people were excluded because they indicated that they were currently unemployed or unable to work. Thus, our final sample consisted of 327 participants, of which 78 people completed the survey before restrictions were announced and telework became mandatory whenever possible (i.e., before 19^th^ of March, 2020; named “Group 1” in the following) and 249 people after the restrictions were implemented (i.e., after 19^th^ of March, 2020; named “Group 2” in the following). Twenty-six percent were male, 74% female and one person indicated “diverse” as gender. Average age was 35.28 years (*SD* = 10.38). On average, participants reported working 32.35 h per week (*SD* = 13.13) and 36% indicated having leadership responsibilities. Participants worked in a variety of industries, the most common being health care and social services (30%), education (14%), and public administration (11%).

#### Measures

*Telework* was measured by asking participants if and how many hours they were working from home. Rather than dichotomizing individuals into low- or high-frequency telecommuting groups, we assessed the hours per week that participants telecommute as this provides more detailed information (see Allen et al., 2015).

*Perceived disadvantages of telecommuting* were assessed with ten items, based on the telecommuting literature and previous results (e.g., Eddleston & Mulki, 2017; Greer & Payne, 2014). Participants were asked to rate to what extent they would agree with different disadvantages of telework on a five-point scale ranging from 1 (*strongly disagree*) to 5 (*strongly agree*). These ten potential disadvantages were: “…no personal contact with colleagues”, “…fewer opportunities to exchange information”, “…blurring the boundaries between work and private life”, “…tendency to work more”, “…feeling socially isolated”, “…coordination with other colleagues is more difficult”, “…difficulties in switching off from work”, “…less time with the leader”, “…fewer chances of career advancement”, and “…distraction by things at home (e.g., children)”. As we had no a priori assumptions of the underlying dimensionality of these items, we decided to first collapse them into a unidimensional scale. Examining the internal consistency of this scale showed a low corrected item-total correlation for the item “tendency to work more” (less than 0.3; Field, 2009) and an inconsistent correlation pattern with the other items. Therefore, we removed this item, which was supported by the results of the confirmatory factor analyses (CFAs): A CFA for a one-factor model with all items revealed a rather poor fit (χ^2^ [35] = 192.77, RMSEA = 0.12, CFI = 0.77) but without the item “tendency to work more”, the data approximation was significantly better (χ^2^ [27] = 123.92, RMSEA = 0.11, CFI = 0.85; Satorra-Bentler scaled Δχ^2^ = 68.34, Δtest scaling correction = 1.17, Δdf = 8, p < 0.001) but not as good as expected according to rule-of-thumb cutoff criteria (Schermelleh-Engel et al., 2003). All CFAs were calculated in Mplus using maximum likelihood estimation with robust standard errors (MLR) that are robust to non-normality (Muthén & Muthén, 1998–2017). An inspection of the modification indices suggested that the parameters of five covariances between error variables should be freed to improve the model fit. These five covariances were theoretically plausible (personal contact, exchange and coordination with colleagues; blurring of boundaries and switching off from work; time with leader and career advancement; distraction at home and blurring boundaries). Allowing these five error covariances, improved the model fit (χ^2^ [22] = 39.17, RMSEA = 0.05, CFI = 0.97; Satorra-Bentler scaled Δχ^2^ = 80.42, Δtest scaling correction = 1.21, Δdf = 5, p < 0.001). All factor loadings were significant (*p* < 0.001). The overall nine-item-scale without the item “tendency to work more” showed a good reliability (α = 0.81).

### Results

#### Preliminary Analyses

Means, standard deviations, and intercorrelations among the variables are presented in Table 1. With regard to the sample characteristics, the two groups did not differ in gender (χ^2^ [2, *n* = 327] = 0.79, *p* = 0.673), or working hours per week (*M*_Group 1_ = 31.63, *SD*_Group 1_ = 19.92, *M*_Group 2_ = 32.58, *SD*_Group 2_ = 10.16; *t*[89.88] = -0.41, *p* = 0.684). The only difference was found regarding participants’ age with participants in Group 1 being slightly younger (*M* = 32.55, *SD* = 9.07) than participants in Group 2 (*M* = 36.14, *SD* = 10.64; *t*[147.12] = -2.92, *p* = 0.004).

**Table 1:** Means, Standard Deviations, Reliabilities, and Correlations among the Study Variables in Study 1

Variable	*M*	*SD*	1	2	3	4	5
1. Age	35.28	10.38	-				
2. Gender	-	-	.34^***^	-			
3. Group	-	-	.15^**^	.04	-		
4. Telework (hours per week)	16.44	13.84	.17^**^	.09	.25^***^	-	
5. Perceived Disadvantages	3.24	.79	-.15^**^	-.04	.17^**^	.02	(.81)

*N* = 327. Reliability coefficients are reported along the diagonal

Gender: 1 = female, 2 = male, 3 = diverse

Group: 1 = Group 1 (before COVID-based restrictions), 2 = Group 2 (after COVID-based restrictions)

^*^p < .05, ^**^p < .01, ^***^p < .001

Analyses of the group differences showed that the same number of participants in both groups used telework (86% in both groups; χ^2^ [1, *n* = 327] = 0.000, *p* = 0.992). However, as expected, participants in Group 2 during the COVID-19 pandemic worked more from home (*M* = 18.38 h per week, *SD* = 14.50) and almost twice as many hours as participants in Group 1 before the COVID-19 pandemic (*M* = 10.24 h per week, *SD* = 9.08; *t*[208] = -5.90, *p* < 0.001). Participants in Group 1 agreed less with the disadvantages than participants in Group 2 (*M*_Group 1_ = 3.00, *SD*_Group 1_ = 0.73, *M*_Group 2_ = 3.31, *SD*_Group 2_ = 0.79; *F*(1, 325) = -3.08, *p* = 0.002).

#### Hypothesis Testing

To test our hypothesis, we conducted moderated regression analyses with the SPSS macro PROCESS using 5,000 bootstrap samples and the standardized independent variable telework (cf. Hayes, 2018). We used the standardized variable to compute the interaction term to reduce multicollinearity. Table 2 shows the results of the moderated regression analysis. The amount of telework was negatively associated with perceived disadvantages (*b* = -0.68, *SE* = 0.27, *p* = 0.013), showing that the more participants teleworked, the less disadvantages they perceived. As already confirmed in the group comparisons, the group variable had a significant influence on the assessment of the perceived disadvantages (*b* = 0.45, *SE* = 0.12, *p* < 0.001): People in Group 2 perceived more disadvantages than people in Group 1.

**Table 2:** Results of the Moderated Regression Analysis in Study 1

	Perceived Disadvantages *b (SE)*
Intercept	2.40***
Telework (hours per week)	-.68*
Group	.45***
Telework X Group	.35*
*R*^*2*^	.05**
Δ*R*^*2*^	.02*

Unstandardized coefficients reported

Group: 1 = Group 1 (before COVID-based restrictions), 2 = Group 2 (after COVID-based restrictions)

Δ*R*^*2*^ refers to the change in explained variance attributable to the inclusion of the interaction term

^†^*p* < .10, ^*^p < .05, ^**^p < .01, ^***^p < .001

**Fig. 2:**
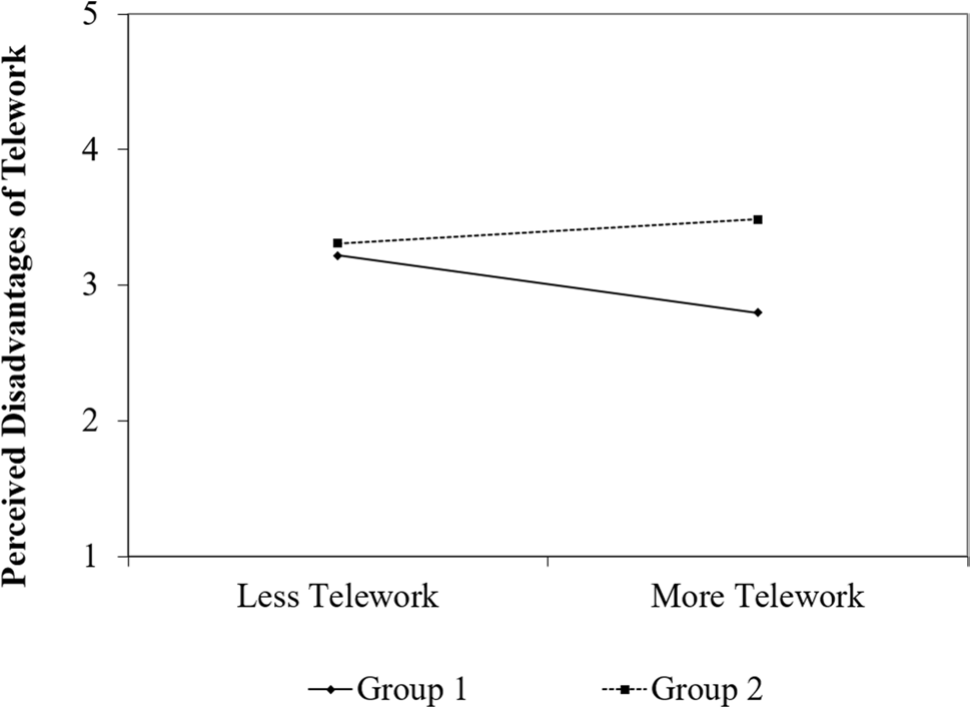
Simple Slopes with values + / − 1 SD in Study 1

The interaction between telework and group in predicting perceived disadvantages was significant (*b* = 0.35, *SE* = 0.14, *p* = 0.014). Figure 2 shows that participants in Group 1 perceived less disadvantages the more telework they did (*b* = -0.33, *SE* = 0.13, *p* = 0.015), whereas in Group 2 the amount of telework was not associated with the perception of disadvantages (*b* = 0.02, *SE* = 0.05, *p* = 0.630). Hence, our hypothesis was supported.

### Exploratory Analyses

Although our hypothesis focused on a unidimensional scale of perceived disadvantages of telecommuting, the CFA results indicate that an overall scale may be too general (e.g., error covariances seem to indicate that a one factor model might not represent the data best). We therefore also conducted an exploratory factor analysis (EFA). This procedure, which deviates from the usual way (first EFA, then CFA), is due to the fact that we initially assumed a one-dimensional structure in accordance with the hypothesis, but the results of the CFAs caused us to question this and we then computed an EFA for exploratory purposes. The Kaiser–Meyer–Olkin measure verified sampling adequacy for the analysis (KMO = 0.84) and the Bartlett’s test of sphericity (χ^2^ (36) = 789.83, p < 0.001) indicated that correlations between items were sufficiently large (Field, 2009). An exploratory factor analysis (Principal Axis Factor method with Varimax rotation, which is recommended for exploring datasets, Yong & Pearce, 2013) revealed two components to have an eigenvalue over the Kaiser’s criterion of 1 and in combination explained 40% of the variance, which was supported by the scree plot. The items that cluster on factor 1 represent *isolation and less contact* (“no personal contact with colleagues”, “fewer opportunities to exchange information”, “feeling socially isolated”, “coordination with other colleagues is more difficult”). The item "feeling socially isolated" showed loadings on both factors. Due to the content fit, however, we assigned it to factor 1. The items that cluster on factor 2 represent *problems with work-life-balance* (“blurring the boundaries between work and private life”, “difficulties in switching off from work”, “fewer chances of career advancement”, “distraction by things at home”). The factor loadings after rotation revealed that the item “less time with the leader” had low cross-loadings on both factors which is why we excluded the item from the subsequent analyses. To validate the factorial model derived from the EFA, we run CFAs with the eight remaining items. A CFA for a one-factor model with all eight items loading on one factor revealed a significantly poorer fit (χ^2^ [20] = 101.56, RMSEA = 0.11, CFI = 0.85) than for the two-factor model (χ^2^ [19] = 48.21, RMSEA = 0.07, CFI = 0.95; Satorra-Bentler scaled Δχ^2^ = 46.92, Δtest scaling correction = 1.29, Δdf = 1, p < 0.001). For the two-factor model, all factor loadings were significant (*p* < 0.001). However, the scale reliabilities were only acceptable (factor 1: α = 0.78; factor 2: α = 0.65). We then conducted separate regression analyses for the two factors discovered by the EFA.

For the first factor *isolation and less contact,* the amount of telework was not significantly related to these perceived disadvantages (*b* = -0.61, *SE* = 0.34, *p* = 0.071) but participants in Group 2 perceived more such disadvantages (*b* = 0.51, *SE* = 0.14, *p* < 0.001). The interaction between telework and group in predicting the disadvantages of *isolation and less contact* was not significant (*b* = 0.31, *SE* = 0.18, *p* = 0.074).

For the second factor *problems with work-life-balance,* the amount of telework (*b* = -0.80, *SE* = 0.31, *p* = 0.009) as well as the group membership (*b* = 0.45, *SE* = 0.13, *p* = 0.001) significantly predicted perception of these disadvantages and the interaction term was also significant (*b* = 0.41, *SE* = 0.16, *p* = 0.011). Again, people in Group 1 perceived fewer disadvantages regarding *problems with work-life-balance* the more they teleworked (*b* = -0.39, *SE* = 0.15, *p* = 0.010) but not people in Group 2 (*b* = 0.02, *SE* = 0.05, *p* = 0.707).

### Discussion of Study 1

Study 1 offers initial support for our hypothesis that the amount of telework is negatively related to perceived disadvantages of telework for individuals before the COVID-based restrictions (Group 1), who were most likely to have chosen to telework voluntarily. For those individuals who teleworked due to the COVID-19 circumstances (Group 2), the amount of telework was not related to perceived disadvantages. The exploratory analyses of the two subscales showed that this mainly relates to the perception of disadvantages in terms of work-life balance but less with regard to isolation and less contact with colleagues.

A limitation of this study is that we did not explicitly measure whether individuals had chosen to telework voluntarily or not, but we only concluded this based on the date of completing the survey which was related to more or less mandatory telework due to the COVID-situation. Therefore, we conducted Study 2 in which we explicitly asked participants about the extent to which telework was voluntary in their organization.

## Study 2

### Method

#### Participants and Procedure

Participants were recruited in Germany via the online platform Clickworker. For their participation in the 15-min study, participants received a small compensation of about 2.5 euros. A total of 250 people completed the online questionnaire. We excluded those participants who failed to correctly answer the attention checks or who finished the questionnaire in less than 50% of the median completion time. Thus, the final sample size was *N* = 220 with 59% men and 41% women. The mean age was *M* = 39.28 (*SD* = 11.09). Participants indicated an average of 34.71 h worked per week (*SD* = 8.16) and 25% reported leadership responsibilities. Participants worked in a variety of industries, with information and communications (19%), manufacturing (e.g., engineering; 13%), and professional, scientific, and technical services (9%) cited most frequently.

#### Measures

*Telework* was measured by asking participants if and how many hours they were working from home. This time, we asked about the amount of telework during regular work hours and outside of regular work hours and used the sum of these hours because we were interested in the total number of hours per week that people teleworked.

*Perceived disadvantages of telecommuting* were assessed with the same items as in Study 1 and participants rated their agreement with these different disadvantages on a five-point scale (1 = *strongly disagree* to 5 = *strongly agree*). Again, as in Study 1, a CFA of a nine-item scale without the item “tendency to work more” revealed a better fit (χ^2^ [27] = 146.76, RMSEA = 0.14, CFI = 0.76) than with all ten items (χ^2^ [35] = 188.80, RMSEA = 0.14, CFI = 0.72; Satorra-Bentler scaled Δχ^2^ = 42.17, Δtest scaling correction = 1.39, Δdf = 8, p < 0.001). As in Study 1, the fit improved when five theoretically plausible error terms were allowed to covary (personal contact, exchange, feeling of social isolation and coordination with colleagues; blurring of boundaries and switching off from work; blurring of boundaries and switching off from work; χ^2^ [22] = 36.08, RMSEA = 0.05, CFI = 0.97; Satorra-Bentler scaled Δχ^2^ = 93.40, Δtest scaling correction = 1.48, Δdf = 5, p < 0.001). All factor loadings were significant (*p* < 0.001). Thus, as in Study 1, we used this scale with nine items (α = 0.83) for testing our hypothesis.

Participants’ perception of *voluntariness of telework* was measured by asking “To what extent can employees in your company choose to telework voluntarily? (This is about your subjectively perceived voluntariness)” (rated on a five-point scale from 1 = *not at all voluntary* to 5 = *absolutely voluntary*). We recoded this scale so that low scores depicted strong voluntariness and high scores depicted low voluntariness, similar to the group variable in Study 1.

### Results

Table 3 shows the means, standard deviations, and intercorrelations among the variables. We used the same analytic procedure to test our hypothesis as in Study 1: We standardized the independent and moderator variables before computing the interaction term. The results of the moderated regression analysis can be found in Table 4. In line with Study 1, the amount of telework was negatively associated with perceived disadvantages (*b* = -0.11, *SE* = 0.57, *p* = 0.051). However, the relationship was not significant. Voluntariness of telework did not predict the perception of disadvantages (*b* = -0.05, *SE* = 0.57, *p* = 0.355).

**Table 3:** Means, Standard Deviations, Reliabilities, and Correlations among the Study Variables in Study 2

Variable	*M*	*SD*	1	2	3	4	5
1. Age	39.28	11.09	-				
2. Gender	-	-	-.11	-			
3. Voluntariness of Telework	3.11	1.23	-.06	-.00	-		
4. Telework (hours per week)	22.70	17.83	.12	-.08	-.31***	-	
5. Perceived Disadvantages	3.30	.80	-.03	-.00	-.04	-.10	(.83)

*N* = 220. Reliability coefficients are reported along the diagonal

Gender: 1 = female, 2 = male, 3 = diverse

^*^p < .05, ^**^p < .01, ^***^p < .001

**Table 4:** Results of the Moderated Regression Analysis in Study 2

	Perceived Disadvantages *b (SE)*
Intercept	3.34***
Telework (hours per week)	-.11^†^
Voluntariness of Telework	-.05
Telework X Voluntariness of Telework	.12*
*R*^*2*^	.04^†^
Δ*R*^*2*^	.02*

Unstandardized coefficients reported

Δ*R*^*2*^ refers to the change in explained variance attributable to the inclusion of the interaction term

^†^*p* < .10, ^*^p < .05, ^**^p < .01, ^***^p < .001

**Fig. 3:**
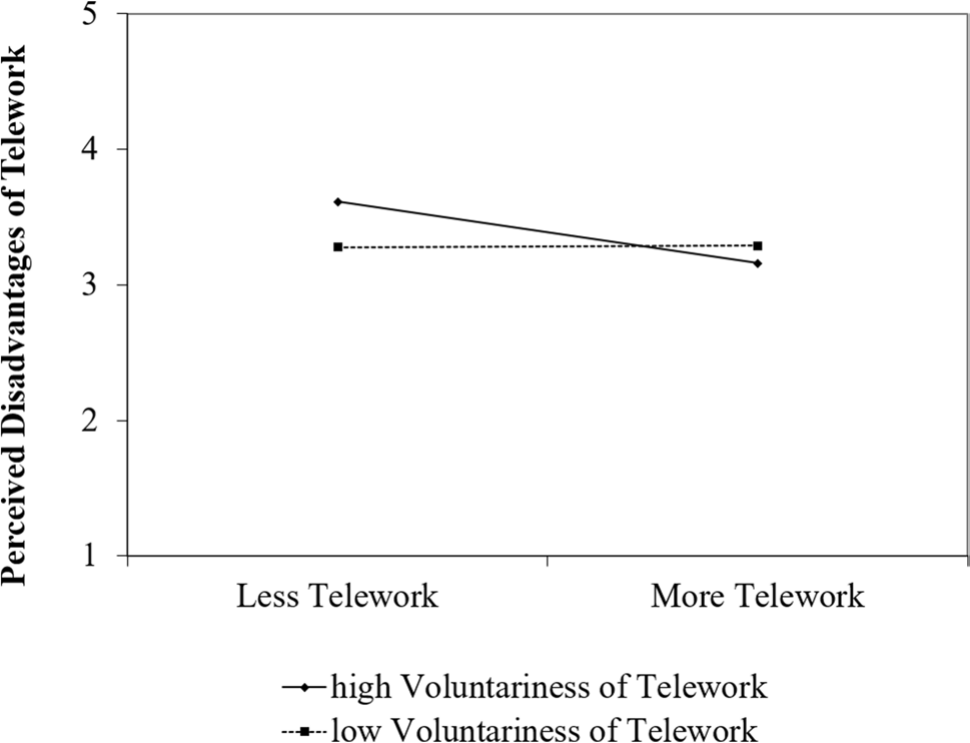
Simple Slopes with values + / − 1 SD in Study 2

The interaction between telework and voluntariness of telework significantly predicted perceived disadvantages (*b* = 0.12, *SE* = 0.06, *p* = 0.038). Simple slope analysis (see Fig. 3) revealed that participants with high voluntariness perceived less disadvantages the more telework they did (*b* = -0.23, *SE* = 0.08, *p* = 0.007), whereas for participants with low voluntariness the amount of telework was not associated with the perception of disadvantages (*b* = 0.01, *SE* = 0.08, *p* = 0.949). Thus, our hypothesis was again supported.

### Exploratory Analyses

As in Study 1, we also examined the factor structure of the nine items of perceived disadvantages of telecommuting in an exploratory EFA (Principal Axis Factor method with Varimax rotation: KMO = 0.79; Bartlett’s test of sphericity χ^2^ (36) = 655.89, *p* < 0.001). The results again revealed two components with eigenvalues over 1 which together explained 45% of the variance. This was supported by the scree plot. As in Study 1, the items “feeling socially isolated” and “less time with the leader” showed cross-loadings on both factors. Similar to Study 1, we classified the item “feeling socially isolated” to the first factor based on its content and removed the item “less time with the leader”. In addition, the item “no personal contact with colleagues” also showed some cross-loadings but with higher loadings on factor 1 which is why we have assigned it to this factor in accordance with its content and Study 1. As in Study 1, items representing *isolation and less contact* clustered on factor 1 (“no personal contact with colleagues”, “fewer opportunities to exchange information”, “feeling socially isolated”, “coordination with other colleagues is more difficult”) while the items that load on factor 2 represent disadvantages regarding the *work-life-balance* (“blurring the boundaries between work and private life”, “difficulties in switching off from work”, “fewer chances of career advancement”, “distraction by things at home”). A CFA of these eight items showed that a one-factor model (χ^2^ [20] = 133.93, RMSEA = 0.16, CFI = 0.73) fit the data worse than a two-factor model (χ^2^ [19] = 96.45, RMSEA = 0.14, CFI = 0.82; Satorra-Bentler scaled Δχ^2^ = 35.87, Δtest scaling correction = 1.30, Δdf = 1, p < 0.001). Consistencies for both scales were sufficient (factor 1: α = 0.76; factor 2: α = 0.74). As in Study 1, we ran exploratory analyses with the two subscales.

For perceived disadvantages regarding *isolation and less contact* (factor 1), we found that neither the amount of telework (*b* = -0.07, *SE* = 0.07, *p* = 0.328) nor voluntariness predicted these disadvantages (*b* = -0.10, *SE* = 0.07, *p* = 0.134) and the interaction was not significant (*b* = 0.12, *SE* = 0.07, *p* = 0.070). An exploratory inspection of the simple slopes showed an insignificant but negative relation between the amount of telework and the perception of disadvantages regarding *isolation and less contact* for participants with high voluntariness (*b* = -0.18, *SE* = 0.10, *p* = 0.059) but a positive and insignificant relation participants with low voluntariness (*b* = 0.05, *SE* = 0.09, *p* = 0.550).

For the perceived disadvantages regarding *problems with work-life-balance* (factor 2), the amount of telework negatively related to the perception of these disadvantages (*b* = -0.16, *SE* = 0.06, *p* = 0.014). Voluntariness did not predict these perceived disadvantages (*b* = 0.03, *SE* = 0.06, *p* = 0.686) and the interaction was not significant (*b* = 0.11, *SE* = 0.06, *p* = 0.087). Again, an exploratory inspection of the simple slopes showed a relation between amount of telework and these disadvantages for participants with high voluntariness (*b* = -0.27, *SE* = 0.09, *p* = 0.005) but no relation for participants with low voluntariness (*b* = -0.05, *SE* = 0.09, *p* = 0.550).

### Discussion of Study 2

Results of Study 2 corroborated the findings from Study 1 that individuals who teleworked more perceived fewer disadvantages, but only if they reported a high level of voluntariness regarding their telecommuting arrangement. For individuals for whom telework was less voluntary, the amount of telework was not associated with perceptions of disadvantages. Interestingly, in Study 2, neither the amount of telecommuting, i.e., how many hours the participants worked from home, nor the voluntariness of telecommuting alone were significantly associated with the perception of disadvantages. This shows that it is not the amount of telework per se that is related to the perceived disadvantages, but that the voluntariness of such a work form is decisive and determines if there is a relation between amount of telework and disadvantages or not. The exploratory analyses for the two subscales revealed similar patterns (albeit with insignificant interactions) for both perceived disadvantages regarding *isolation/less contact* and *problems with work-life balance* and simple slopes matching the pattern of the analyses with the full scale.

## General Discussion

During the COVID-19 pandemic, a challenge for organizations and leaders is to implement teleworking and to deal with potential concerns that employees may have regarding this form of work. Our results revealed that individuals who can choose to telework voluntary reported fewer disadvantages the more telework they did. However, the amount of telework was not related to reduced perceptions of disadvantages for those individuals who reported low voluntariness regarding (as was the case in many organizations during the COVID-19 pandemic).

These findings are not only of relevance during the COVID-19 pandemic, but also offer a general starting point for organizations and leaders on how to introduce telework. Our study thus responds to calls for further research on voluntary vs. involuntary telework (Bartel et al., 2011; Beauregard et al., 2019) and can contribute to a better understanding of the different effects that mandatory vs. self-chosen telework can have as a boundary condition to effective telework. Previous studies show that the extent to which individuals have control and autonomy over their work tasks (Golden & Veiga, 2005) and the flexibility in choosing when work activities are performed (Golden et al., 2006) influence the relationship between telework and various outcomes, such as work-family conflict. Although tentative and in need of replication, our findings suggest that not only autonomy regarding the execution of the job (for example, with regard to work tasks or working hours) is important, but also whether employees are free to choose the number of hours they want to telework.

We based our theoretical reasoning on cognitive dissonance theory (Festinger, 1957) to explain how the perception of possible negative aspects of telecommuting is reduced by offering this form of work to employees voluntarily. We theorized that employees who voluntarily work from home suppress negative aspects in order to reduce cognitive dissonance. Future studies could explore this aspect further by explicitly capturing the cognitive dissonance that arises when employees voluntarily choose to telework but face associated disadvantages (e.g., work-life conflicts). It would also be interesting for future research to further explore our findings with respect to the job demands control model (e.g., Karasek, 1979, 2004). For example, one question would be to what extent the voluntary nature and control over this work form can buffer negative outcomes of specific demands. Even though the results of the explorative analyses in the first study suggest that especially the relationship between amount of telework and negative aspects related to work-life balance is moderated by the voluntary nature of telework, these results are still tentative and should be further explored by additional studies.

In Study 1, we adopted a natural intervention, which is a good means of examining variation in variables that are otherwise difficult to manipulate. We therefore chose the date of completing the questionnaire before and after the 19^th^ of March 2020 as an operationalization for the voluntariness of telework. In Germany, where the study was conducted, mandatory telework was introduced that week in many organizations and therefore we assume that participants were required to telework as of that date. However, it could be that some people in Group 2 (during the pandemic) also liked telework and had chosen it voluntarily. For this reason, we conducted a second study in which we explicitly asked participants about their subjectively perceived voluntariness of telework. The results of Study 2 corroborate those from Study 1 and therefore confirm our assumption that it is the voluntariness of telework that makes a difference. Nevertheless, it could be that other factors have an influence on the perception of disadvantages of telework. For example, the family situation (e.g., family support or interruption, care of small children, care of elderly relatives), the financial situation (e.g., fear of job loss, salary cuts due to short-time work or loss of bonus payments), and the work situation at home (e.g., technical conditions, ergonomic conditions) are important. Aspects of the work itself, for example whether the work task is difficult to implement when teleworking such as tasks which require high levels of interdependence, could also have an influence. Future studies should examine these factors and their influence on the perception of disadvantages of telework.

It should also be noted that teleworking had some specific features in the context of the COVID-19 pandemic. It was a change that affected many employees together and not only individual colleagues. It may be that the effects differ if not everyone in a team performs telework, but only some employees (i.e. when no norms for telecommuting exist, see e.g., Gajendran et al., 2015). During the COVID-19 pandemic, when suddenly all (or most) colleagues were working from home, new communication routines and methods could be developed within the team and solutions could be worked out together, which may have increased the perception of potential positive consequences of telework (see Gajendran & Harrison, 2007). Indeed, Golden and Eddleston (2020) found that employees working in teams where telework was highly normative (as compared to work groups where this was rather uncommon), experienced more positive consequences, such as promotions. If, on the other hand, only individual employees work from home and ways to stay in touch with them need to be found, it could be that the disadvantages related to the team and colleagues (such as feelings of isolation, poorer communication and relationships with colleagues and superiors) are of particular importance.

### Practical Implications

From a practical perspective, the present findings illuminate tangible approaches that organizations and leaders can adopt to minimize potential negative effects of telecommuting. Raising leaders’ and employees’ awareness and providing an understanding of the possible adverse consequences of (involuntary) telework would be a first step. This could be done, for example, by explaining the reasons for the implementation of telework. Second, even when organizations request telework as standard form of working, employees can still be given as much control as possible, for example in choosing the days on which they work remotely. One form of work that is increasingly being implemented in companies are blended working arrangements (e.g., Wörtler et al., 2020). Such working arrangements allow employees to decide flexibly when and how long they work (time-independent working) and from where they perform their work (location-independent working) (e.g., Van Yperen et al., 2014). This means that employees can decide for themselves whether they work from home or at the employer’s site, and not within the framework of a rather rigid telework program, but flexibly adapted to the respective needs and work tasks. This enables employees to work from home on a voluntary basis if they want to and if they think it is useful and this voluntary nature of the work arrangement should lead to the perception of fewer disadvantages of telework, as our results show.

### Limitations and Further Research

One limitation of the study is its cross-sectional design, which constrains conclusions about causality. However, the postulated effects are consistent with theory and previous findings. Nevertheless, it would be desirable for future research to examine the proposed effects in designs that allow causality testing, such as experimental longitudinal designs. A unique feature of Study 1 is its natural occurring massive change in telework due to the pandemic. However, this also entails some limitations, such as the lack of a control group as well as no randomization. Future studies applying experimental research designs for instance in organizations in which telework is newly introduced would be desirable to verify our results.

Another limitation is the use of single-source data, which involves the risk of common method bias. To deal with potential issues resulting from using the same source, we took several steps, such as assuring respondents’ anonymity (e.g., Podsakoff et al., 2003).

In addition, the use of a crowdsourced sample as in Study 2, is sometimes seen as a potential limitation. Even though platforms such as Clickworker or MTurk show good data quality and are frequently used in research, there is a risk that participants will not attentively complete the questionnaire (Cheung et al., 2017). To minimize the risk, we incorporated attention checks as well as excluded participants who answered the questionnaire in an unrealistically short time.

Finally, in Study 2, we measured voluntariness by asking participants a single question about their subjective perception of whether their organization would give employees choice over choosing telework or not. Future studies may use the question of whether or not it was the participants’ own decision to telework. An alternative may be to conduct studies in multiple organizations with different policies or cultures of telework, ranging from restricting telework to very few jobs to enforcing telework for all employees.

Future research may also include personal characteristics such as an employee’s need strength. Van Yperen and colleagues (2014) found that the perceived effectiveness of blended working was moderated by the need for autonomy and the need for relatedness. Similarly, O’Neill and colleagues (2009) discussed personality characteristics such as sociability as factors determining the success of telework. So, future studies may explore if the moderating effect of voluntariness could be further moderated by the need for autonomy and if the negative consequences around loneliness could be accentuated by the need for relatedness or traits like sociability.

Future research may also look into the endorsement of teleworking by organizations, leaders and employees. This may be especially important in situations in which the presence of employees has traditionally be enforced even when it was not required (as is the case in production facilities operating heavy machinery). Such forced presence can be assumed to frustrate employees' psychological need for autonomy, with its documented negative outcomes on health and employee growth (see Vansteenkiste & Ryan, 2013).

Another avenue for future research is suggested by the exploratory analyses regarding the kind of perceived disadvantages. These analyses indicated two main aspects – namely less contact and resulting isolation on the one hand and difficulties with respect to work-life balance on the other. The pattern of these exploratory analyses matched our main results but mostly with insignificant interactions. This may be due to the relatively low internal consistencies of the subscales which may be improved in future research by adding specifically formulated items to each dimension.

Finally, the two studies were conducted in one country (Germany) only, which may limit the generalizability to other countries and cultures. Hence, a promising avenue for future studies would be to examine cultural influences on the perception of disadvantages of telework and on the moderating role of the voluntariness of the telework arrangement. Such future studies in other contexts would also rule out that our results for voluntariness in Study 2 are also affected by the COVID-related restrictions themselves, i.e. by the fact that at least for some participants who had indicated to telework voluntarily, they had also been forced to do so because of the pandemic.

## Conclusion

Allen et al. (2003) stated “the question of whether telecommuting is a good or bad way of designing work is probably not as important as questions of how to design and implement telecommuting arrangements.” (p. 155–156). In this sense, the present work identifies voluntariness over the form of work as a possible approach to buffering the potentially negative consequences of telework perceived by employees. It is important to emphasize that the exceptional situation during the pandemic forced telework under simultaneous national and international containment measures and therefore conclusions about telework in general are only possible to a limited extent. However, we hope that our findings offer possible starting points for organizations and leaders and serve as a basis for further research.

## Data Availability

The data of both studies is available at the open science framework under: https://osf.io/2pehz/?view_only=978de6a1d86e4732a3251580230b856e
